# Pressure-Induced Polymerization: Addition and Condensation Reactions

**DOI:** 10.3390/molecules26247581

**Published:** 2021-12-14

**Authors:** Fang Li, Jingqin Xu, Yajie Wang, Haiyan Zheng, Kuo Li

**Affiliations:** Center for High Pressure Science and Technology Advanced Research, Beijing 100094, China; fang.li@hpstar.ac.cn (F.L.); jingqin.xu@hpstar.ac.cn (J.X.)

**Keywords:** high pressure, polymerization, topochemical reaction, reaction mechanism, hydrogen transfer

## Abstract

Under pressure of 1–100 GPa, unsaturated organic molecules tend to form covalent bond to each other for a negative enthalpy change, which often produces polymeric materials with extended carbon skeleton. The polymerization reactions typically happen in crystal, which promotes the topochemical process. This review summarized the topochemical polymerization processes of several alkynes, aromatics, and alkynylphenyl compounds, including the critical crystal structures before the reaction, bonding process, and the structure of the products. Secondly, this review also summarized the condensation reaction identified in the polymerization process, including the elimination of small molecules such as NH_3_, etc.

## 1. Introduction

Chemical reactions under high pressure (HP) are often related to high temperature (HT), since, under high pressure, the migration of atoms is hindered, and extra energy is required to overcome the energy barrier to reach the thermodynamic stable state, that is, the minimum point on the potential surface. This is what proceeds in most high-pressure experiments currently. However, much more meta-stable structures are remaining at the local minimums on the potential surface, waiting to be explored. The most famous examples are diamond and its analogues [1,2,3]. The HP-HT synthesis can easily synthesize cubic diamond as it is the thermodynamic stable phase at the P-T condition, but not for its metastable analogues with other sp^3^-C-C connection topologies.

This is similar to the traditional HT solid state reactions and molecular/cluster solution reactions at ambient pressure (AP). The dedicatedly designed solution reactions can drive the small molecules to travel on the potential surface, control the directions, and build up highly complex structures (bottom-up) with complicated physical and chemical functions. To do the same thing under HP and precisely synthesize the required metastable materials, a bottom-up process is also needed, including choosing suitable building blocks such as molecules and/or clusters, and the techniques to connect the building blocks.

**Scheme 1:**
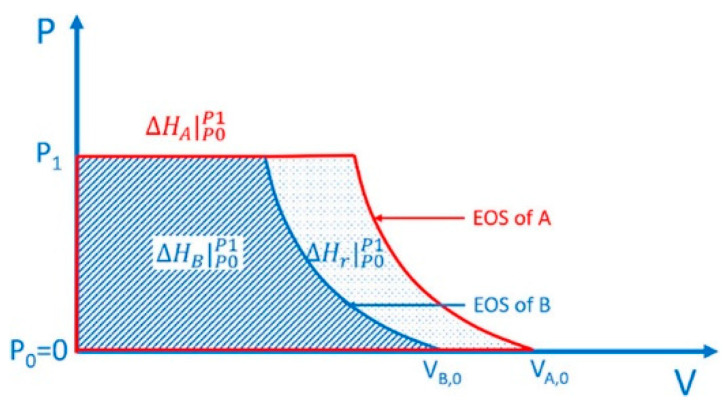
Contribution of external pressure to the enthalpy of a substance equals to the integral of its equation of state on the pressure-axis ($\Delta H_{A}{|}_{P0}^{P1}$, $\Delta H_{B}{|}_{P0}^{P1}$) and the contribution to the enthalpy change of a reaction (A = B) equals to the difference of the integrals ($\Delta H_{r}{|}_{P0}^{P1}$). Reprinted with permission from ref. [4]. Copyright 2021 American Chemical Society.

For HP reaction, as mentioned above, the migration of molecules is hindered, and HP becomes the most important driving force to the reaction. This brings two characteristics. First, thermodynamically, the ΔPV term affects the enthalpy change and free energy change of the reaction significantly, and the reaction with a negative volume change is more preferred. In practice, molecules always tend to polymerize upon compression, which is referred as pressure-induced polymerization (PIP). We have provided a schematic quantitative thermodynamic explanation and discussions on this in a very recent paper and in previous reviews [4,5,6,7,8,9]. The key point is, the contribution of external pressure to enthalpy is the integral of the equation of state of each material on the pressure-axis, and the difference between the integral of product and reactant is the contribution of external pressure to the enthalpy change of the reaction (Scheme 1, [4]). Second, dynamically, the reaction has strong topochemical features, and the relative positions of the building blocks in the crystal structure of the reactant affect the reaction significantly. Here we define “topochemical reaction” as a solid-state reaction with the crystal structure of product similar to that of the reactant, and the atoms in the crystal structure of the product can be clearly related to those in the reactant.

In this paper, we mainly review the recent works of our group on the PIP of molecules. There are already many excellent reports and reviews in this field and related areas [5,6,7,8,9,10,11,12,13], which we are not going to repeat here. Our group mainly focuses on the exploration of carbon-based materials using PIP, and has made great efforts on unrevealing the chemical reaction mechanism of PIP under HP. Here, we wish to give a summary on the reaction mechanism of the investigated PIP, and provide ideas for achieving precise synthesis of materials at the atomic level under HP.

Typically, PIP includes addition polymerization and condensation polymerization. The former mainly appears in the unsaturated molecules. Upon compression, the sample volume and the intermolecular distances decrease, which enhances the intermolecular interaction, and efficiently induces sp/sp^2^-carbon to transform into sp^2^/sp^3^-carbon, sometimes producing crystalline materials. Since it is solid-state reaction, the crystal structure, including molecule arrangement and the interatomic distances, plays a vital role in determining the reaction pathway and the final polymeric structure. We summarized the critical reaction distances (D_C_, at room temperature without thermal correction unless noted) and the factors that can affect the reactions, which will help to regulate reaction route and obtain a desired polymeric material.

For condensation polymerization, monomers react with each other, accompanied by the elimination of some small molecules. The PIP of acetonitrile is a typical example with loss of ammonia [14]. In most cases, the hydrogen bonds and the hydrogen transfer reaction participate as an important role. Under HP, the hydrogen bond is compressed, which could efficiently lower the energy barrier and promote hydrogen transfer. This would subsequently activate the reactant and trigger the PIP. Therefore, the second part of this paper reviews the typical condensation polymerizations and reveals the significance of hydrogen transfer in the condensation reaction for PIP.

## 2. Results

### 2.1. PIP: Addition Reactions

#### 2.1.1. Alkyne and Acetylides

**Figure 1:**
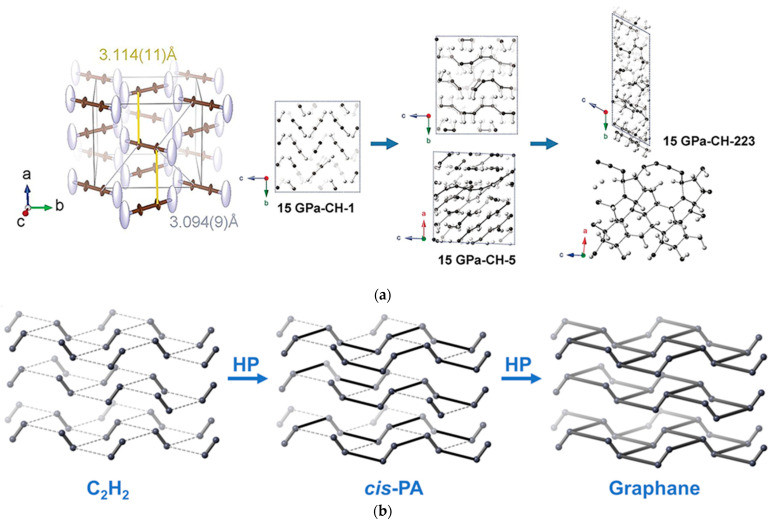
(**a**) (left) Crystal structure of acetylene at 5.7 GPa. The brown and light blue ellipsoids are carbon and deuterium atoms, respectively. The yellow lines indicate the forming of *cis*-PA while the grey for *trans*-PA. (right) Metadynamics simulation results of acetylene under 15 GPa. Reprinted with permission from ref. [23]. Copyright 2017 Angewandte Chemie International Edition; (**b**) PIP of acetylene, via *cis*-PA to graphane. Hydrogen is omitted for clarity.

As the simplest alkyne, acetylene (C_2_H_2_) is an excellent model for the research of PIP. The addition polymerization of acetylene at AP was widely investigated in the last century and Nobel Prize was awarded to the research on conductive polyacetylene (PA). The PIP of acetylene has completely different reaction routes to those under AP. Under HP, acetylene solidifies into crystal and starts to polymerize at 3.5 GPa at room temperature, with the main product confirmed as *trans*-PA [15,16,17,18], whereas, at 77 K, solid acetylene polymerizes at 12 GPa, consequently forming *cis*-PA [19]. Since it is well known that the *trans*-PA is more stable, we need to clarify the reaction mechanism of this selective PIP of acetylene. We used Paris-Edinburgh Press (PE press) as a HP generation apparatus to avoid the photoactivation and facilitates the in situ neutron diffraction [20,21,22]. The crystal structure of deuterated acetylene at the critical reaction pressure of 5.7 GPa was determined by in situ neutron diffraction, from which we found the possible reaction route to produce *cis*-PA [23]. As shown Figure 1a (left), the previous proposed bonding route along a+b or a−b direction will result in *trans*-PA (grey line route) [17,18], while there is another bonding route along the a+c and a−c direction with D_C_ of 3.1 Å (yellow line), which will promote the formation of *cis*-polymer. The fact that anisotropic thermal factors of carbon and hydrogen (deuterium) reach maximum in the a−c plane also supports this bonding route. On the other hand, in the metadynamics simulation, it also shows that the reaction also takes place in the a+c direction, and the formation of *cis*-PA is reproduced (Figure 1a (right)). Additionally, the simulation predicted the production of graphane in a further step of PIP, which was demonstrated by our subsequential experiment. This is a typical example that shows the sp-sp^2^-sp^3^ sequence in the PIP, and also reveals the neighbor-first topochemical rule in understanding the PIP process (Figure 1b).

The acetylide anions such as C≡C^2−^ and HC≡C^−^ were also predicted to polymerize into conjugated extended carbon framework with excellent electrical conductivity under HP [24,25,26,27,28,29]. As the simplest acetylides, CaC_2_ and Li_2_C_2_ were investigated under HP [27,30,31,32,33], and we firstly evidenced their PIP experimentally, which resulted in a remarkable conductivity enhancement,10^7^-fold for CaC_2_, and 10^9^-fold for Li_2_C_2_ [31,32]. After releasing to the atmospheric pressure, due to the extended covalent conjugated C-C bonding, the high conductivity of the recovered products maintains.

**Figure 2:**
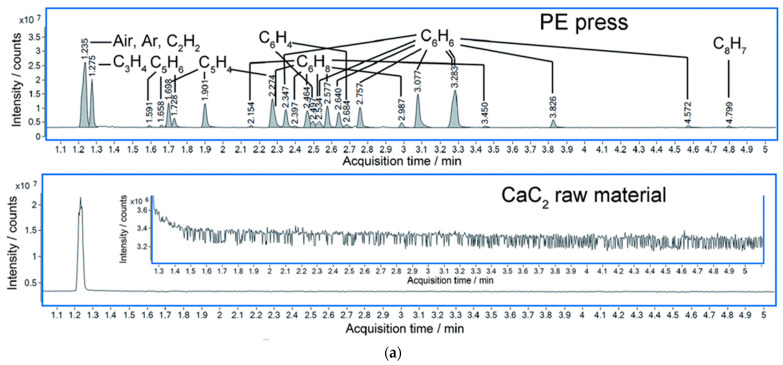

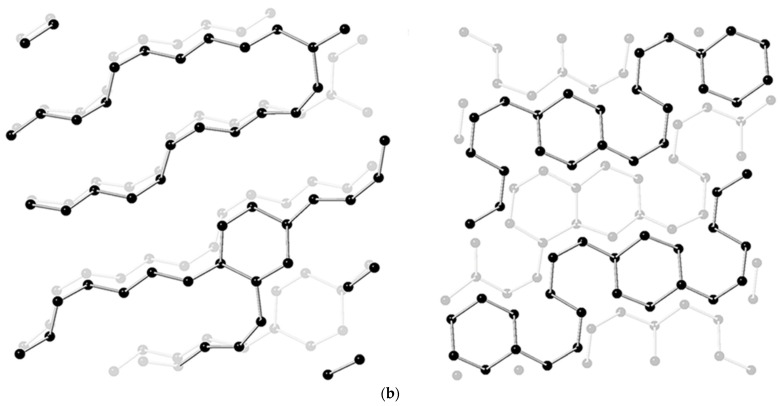
(**a**) Total ion chromatograms (TIC) of the product of CaC_2_ (synthesized by PE press) recovered from 26 GPa and CaC_2_ raw material. (The hydrolysis of the polymerized C_m_^x−^ anions in the recovered sample produced C_2n_H_2n_ according to the chemical reaction equation: Ca_n_C_2n_ + 2nH_2_O = nCa(OH)_2_ + C_2n_H_2n_); (**b**) Simulated chain and ribbon model of CaC_2_ at 30 GPa by metadynamics [31].

We also monitored the crystal structure variation under HP by in situ diffraction and complementary theoretical optimization. CaC_2_ crystallizes in a tetragonal phase at ambient and low pressure, and distorts into a monoclinic phase above ~10 GPa. This accelerates the approaching of neighbored C_2_^2−^, and we estimate that the D_C_ is ~2.9 Å at around 20 GPa [31]. Above 20 GPa at room temperature, diffraction cannot provide enough structural details since CaC_2_ polymerizes into amorphous phase. High resolution gas chromatography mass spectrometry (HR GC-MS) analysis of the hydrated products of the recovered sample provided solid evidence of oligomerization of acetylide. Advanced hydrocarbons were observed besides acetylene. Most of components have the C:H ratio of around 1:1 (Figure 2a), which indicates no obvious disproportionation in the PIP. The slight deviation from 1:1 is likely due to the termination of the free radicals, which was discussed in detail in the work of NaC_2_H [34]. More importantly, benzene (C_6_H_6_) was identified accordingly to HR-GC-MS data. This consists with the reaction sequence of forming chain-belt-sheet as predicted by metadynamics simulation (Figure 2b).

**Figure 3:**
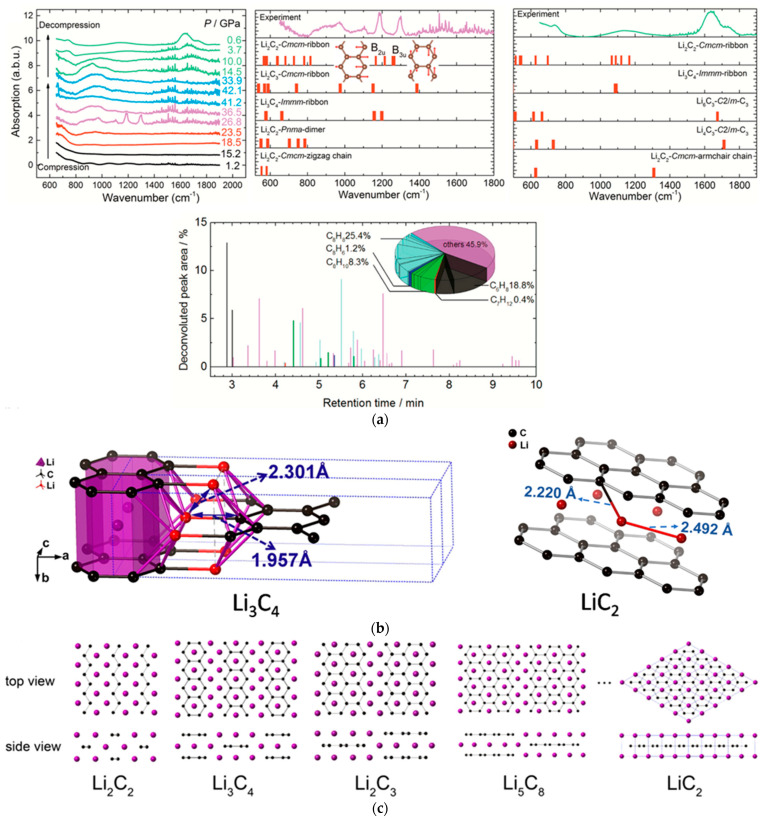
(**a**) The IR spectra of the Li_2_C_2_ (experiment) and the predicted Li-C phases (simulation) under external pressure (above); The relative area of the deconvoluted peaks of the hydrocarbons in the hydrolyzed product (below) in the GC-MS results. The components with a score lower than 90 are counted in “others”. Reprinted with permission from ref. [32]. Copyright 2017 American Chemical Society; (**b**) The crystal structures of Li_3_C_4_ (left) and LiC_2_ (right); (**c**) The predicted crystal structures of Li_n+1_C_2n_ (n = 1, 2, …). The black spheres: C. The purple spheres: Li. Reprinted with permission from ref. [33]. Copyright 2018 American Chemical Society.

By comparing the experimental result of Li_2_C_2_ under high pressure with the theoretical calculated IR spectra, the carbon-ribbon structure in the Li_2_C_2_ polymeric product was identified, and many advanced hydrocarbons in the hydrated products of the recovered sample were detected by GC-MS, which also supports the addition PIP of C_2_^2−^_,_ and the ribbon structures (Figure 3a) [32]. However, different from CaC_2_, there were a portion of products with the C:H ratio significantly deviating from 1:1, which revealed the occurrence of the disproportionation reaction. Compared with the Ca^2+^ in CaC_2_, the Li^+^ cation is more active to diffuse. This leads to disproportion and composition segregation under compression, promoting the formation of Li-rich and C-rich phases. In HPHT experiments, crystalline phase Li_3_C_4_ with zigzag carbon-ribbon structure (lithium polyacenide) and LiC_2_ with intercalation structure (lithium graphenide) were indeed obtained (Figure 3b) [33]. We also predicted a series of lithium polycarbides with a stoichiometric ratio of Li_n+1_C_2n_ (Figure 3c), which share the common feature of zigzag nano-ribbon structure with various width, and LiC_2_ (n = ∞) and Li_3_C_4_ (n = 2) are the two terminal compounds (Figure 3b) [33].

**Figure 4:**
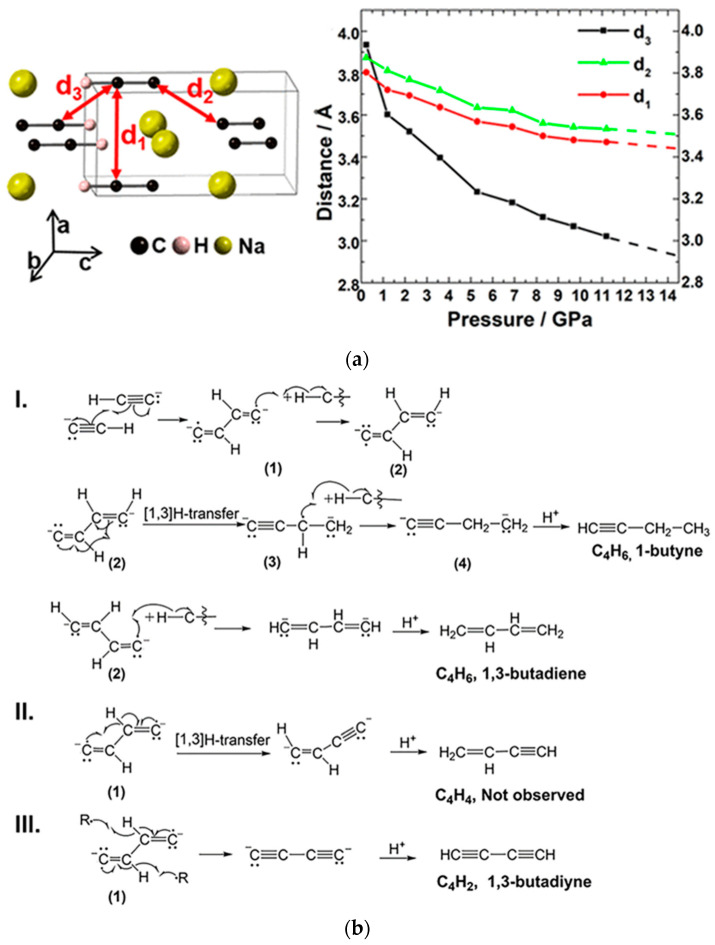
(**a**) Crystal structure of NaC_2_H and the evolution of d_1_, d_2_, and d_3_ as a function of pressure. d_1_, d_2_, and d_3_ represent different intermolecular C···C distance; (**b**) the reaction routes of the NaC_2_H dimerization and the following H-transfer process. Reprinted with permission from ref. [34]. Copyright 2019 American Chemical Society.

As mentioned, acetylene normally reacts at around 5.0 GPa [23], while the polymerization pressures of disubstituted metal acetylides are mostly over 20 GPa (~22 GPa for CaC_2_, and 35 GPa for Li_2_C_2_ [31,32]) due to the repulsion of the electrostatic interaction and spatial blocking of the cations. We also studied the monosubstituted acetylene sodium (NaC_2_H) with only one charge on the carbon group [34]. By the method of in situ spectroscopy and X-ray diffraction (XRD) technology, PIP of NaC_2_H was proved beginning from 14 GPa, which is between that of acetylene and acetylides. According to the crystal structure of NaC_2_H at 11 GPa (the maximum pressure at which we can still have usable diffraction data, Figure 4a (left), the D_C_ is around 3.0 Å (d_3_), and by extrapolation of the d-P curve (Figure 4a (right)) to 14 GPa, the D_C_ was around 2.9 Å. This is similar to that of CaC_2_ [31]. The specific reaction is shown in Figure 4b. This is a free radical addition process. Due to the produced species needing to absorb or lose at most two hydrogen atoms for pairing their two radicals, the hydrolysis products always have a C:H ratio within C_x_H_x±2_ (Figure 4b).

The PIP of C≡C groups should be the simplest PIP reactions. The reactions are likely to follow a free radical route. Though the HP environment prevents us to trap or detect the radicals, the hydrated products of the recovered polyacetylide sample C_n_H_n+2_, and C_n_H_n−2_ etc., provides indirect evidence. The reactions seem to follow the neighbor-first rule, which is a feature of topochemical route. Regardless of the reaction pressure, the bonding share approximately the same D_C_, ~3.0 Å, which is a good reference for other unsaturated groups. The hybridization of carbon in PIP follows a sp-sp^2^-sp^3^ sequence. The PIP can proceed to the end, or stop at half-way, depending on the reactivity of the unsaturated groups and the pressure applied, which provides more controlling factors for synthesizing advanced materials.

#### 2.1.2. Aromatic Compounds

As important building blocks for PIP, aromatic compounds also attract continuously attentions. Since the 1980s, benzene (C_6_H_6_), as the proto model of aromatic systems, has been systematically explored in the high-pressure research [35,36,37,38,39,40,41,42,43,44,45]. In recent years, through optimizing the synthetic conditions (a slow compression/decompression rate), one-dimensional sp^3^ carbon nanothreads were identified from the sample recovered from compressing C_6_H_6_ at room temperature [46,47]. Compared with the traditional sp^2^ nanotube, higher strength and stiffness were expected for the diamond-type nanothreads. Similar results were observed in other aromatics cases, such as pyridine (C_5_H_5_N) [48,49], thiophene (C_4_H_4_S) [50], furan (C_4_H_4_O) [51], and aniline (C_6_H_5_NH_2_) [52,53]. Several reaction routes, including [4+2] addition and 1-1′ addition/coupling, were investigated for benzene, and many models were proposed for the nanothreads [54,55,56]. However, probably due to the diversity of the local structures in the solid sample before reaction, there is still existing considerable confusion about the reaction pathway and mechanism.

**Figure 5:**
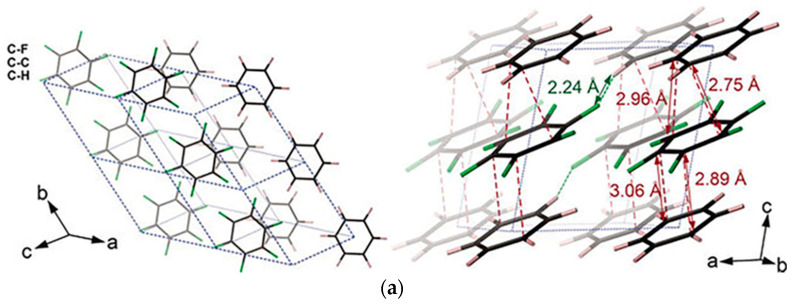

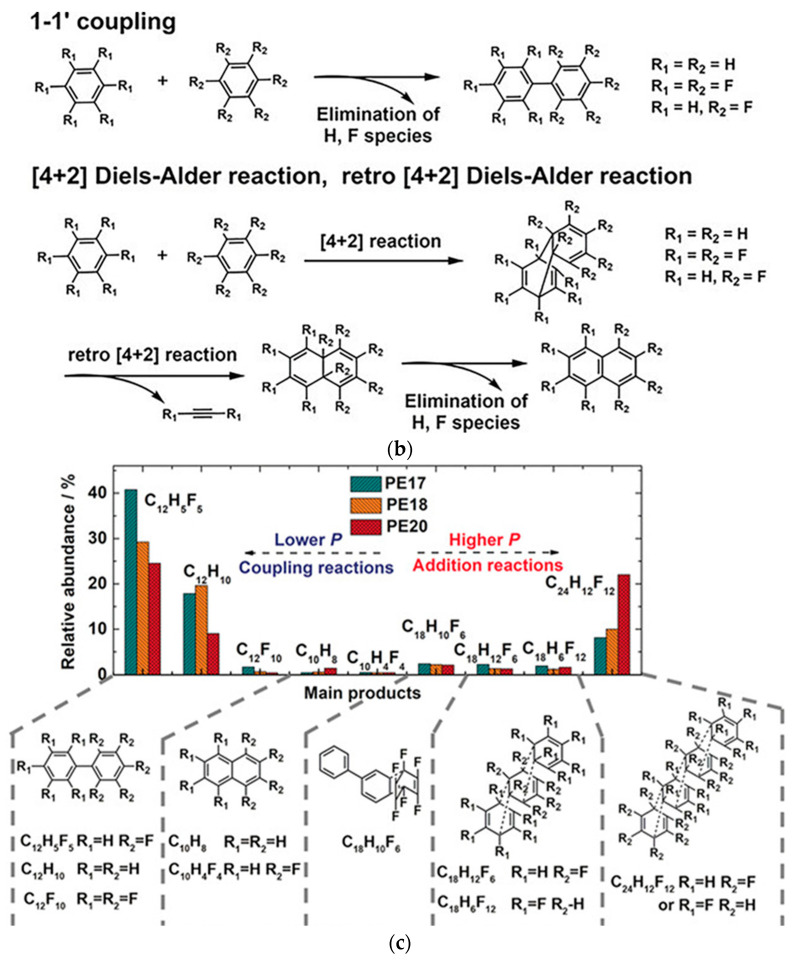
(**a**) Crystal structure of C_6_D_6_-C_6_F_6_ before reaction (20 GPa): (left) viewed perpendicular to (102) and (right) approximately along. Reprinted with permission from ref. [10]. Copyright 2020 Nature; (**b**) Selected elemental reactions of the CHCF cocrystal under high pressure; (**c**) A histogram of the comparison between the relative abundance of major components in different high-pressure samples from GC-MS results. Reprinted with permission from ref. [58]. Copyright 2019 Angewandte Chemie International Edition.

To investigate a model compound with well-defined ordered structure (“monodispersed” local structure), we explored the 1:1 benzene (C_6_H_6_) and hexafluorobenzene (C_6_F_6_) cocrystal [57,58]. It has a strong electrostatic attraction between the two components, which leads to the C_6_H_6_-C_6_F_6_ rings being alternately stacked in column regularly [57]. After releasing from 20 GPa, PIP consequently resulted in a layered structure of H/F substituted graphane. Before reaction occurrence, the structure maintained the column stacking, the column was remarkably titled, and, in fact, the molecules formed closed packed layers (Figure 5a). The D_C_ between C_6_H_6_ and C_6_F_6_ at 20 GPa were decreased to approximately 2.8 Å at room temperature (without thermal correction), reaching the critical reaction distance of previous benzene molecules (2.8~3.0 Å [59]). However, since there are several intermolecular C-C distances closed to D_C_, a [4+2] reaction is probably preferred according to the neighbor-first rule, but is not exclusive (Figure 5b).

Then, we used HR GC-MS to investigate the reaction intermediates to find the primary reaction models in PIP. Taking the standard mass library and the retention time of commercial samples as reference, we concluded that the elemental reactions include Diels-Alder, retro-Diels Alder, and 1-1′ coupling reactions (Figure 5b). By comparing the compositional contents of products recovered from different pressures, we found that product content from the [4+2] Diels-Alder addition route was increased under higher pressure (Figure 5c), rather than the coupling reaction, which is also supported by spectroscopic analysis. Unexpectedly, we did not observe the 1-D nanothread reported in the PIP of benzene, etc., but found an ordered 2-D H/F substituted graphane structure.

After our work, more similar aromatic cocrystal systems were recently reported, including two polycylic 1:1 arene-perfluoroarene cocrystals, naphthalene/octafluorona-phthalene (NOFN), and anthrancene/octafluoronaphthalene (AOFN) [60,61]. Owing to the strong intermolecular reactions between the larger conjugation, their π-π stacked column structure was more stable under compression, and the molecules only tilted slightly with respect to the stacking direction. The D_C_ was along the interplanar centroid-centroid line of polycylic rings, determined as around 2.8 Å for NOFN and 2.7 Å for AOFN, and similar to that of reported aromatics. In contrast, the PIP products are one-dimensional polymers for both NOFN and AOFN.

**Figure 6:**
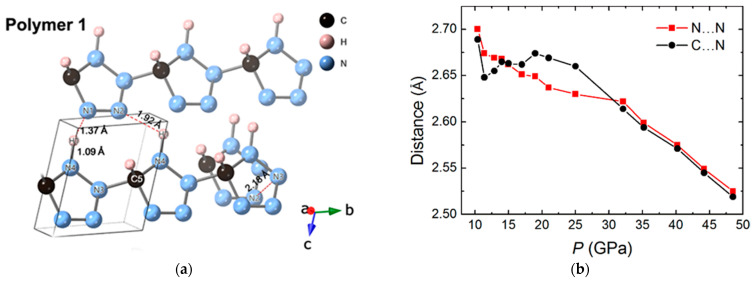
(**a**) Polymeric structure of 1H-tetrazole under 100 GPa, optimized from the structure obtained from the XRD result at 100.3 GPa; (**b**) Closet intermolecular C…N and N…N distance as a function of pressure. Reprinted with permission from ref. [64]. Copyright 2021 Royal Society of Chemistry (RSC) Publishing.

Besides benzene-based derivates, heterocyclic compounds, especially nitrogen-containing cyclic molecules, also attract great scientific attentions [62,63,64]. We investigated the structural evolution and chemical reaction processes of 1H-tetrazole (H_2_CN_4_) [64], a nitrogen-rich compound with a high nitrogen content of 80%, up to 100 GPa for the purpose of creating a novel polynitrogen material with high energy density. Through the in situ synchrotron XRD, an irreversible reaction was demonstrated in the range of 60 to 100 GPa, which was also evidenced by the strong fluorescence signal detected in the recovered sample in Raman spectroscopy. Intermolecular bonding between the C and N atoms from neighbored molecules, respectively, was proposed, according to theoretical investigation (Figure 6a). However, the bonding of nitrogen seems much harder than that of carbon. No chemical reaction proceeds even when the closest intermolecular C…N reaches 2.52 Å at 48.5 GPa, which was much smaller than the D_C_ of unsaturated carbon groups (Figure 6b). The consequence obviously shows that the D_C_ values are highly elemental dependent, probably due to the different electronegativity and/or bonding formation energy.

From our research, we can demonstrate that the PIP of aromatics has various elemental reactions, which can coexist and compete mutually. The intermolecular distances are not far from each other, so the neighbor-first rule may not dominate. By modifying the synthetic conditions, the product may change, which brings great challenges, but also great opportunities.

#### 2.1.3. Alkynlphenyl Systems

**Figure 7:**
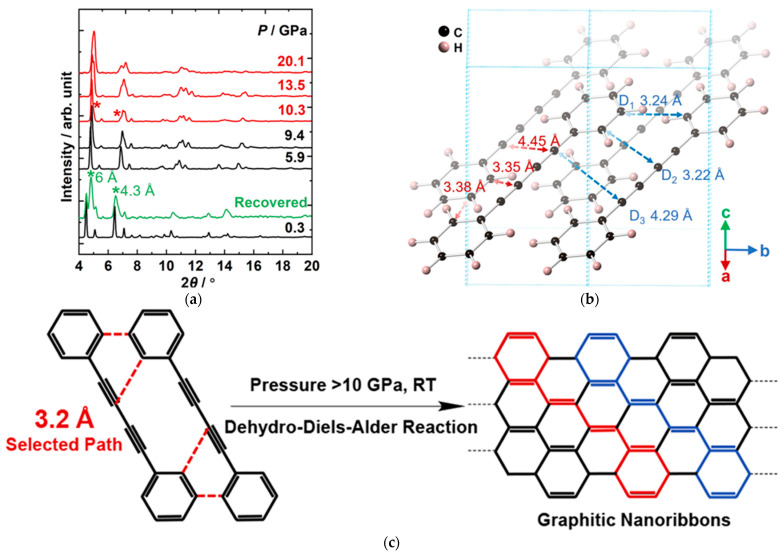
(**a**) Selected in situ XRD of DPB under high pressure. The peaks from the PIP-DPB are marked by the asterisks; (**b**) Crystal structure of DPB optimized by density functional theory (DFT) calculation based on the experimental lattice under 10 GPa. (**c**) Proposed DDA reaction. Reprinted with permission from ref. [65]. Copyright 2020 American Chemical Society.

Generally, the PIP of alkynes proceeds at a relatively lower pressure, while the products often lack long-range ordering at room temperature owing to the thermal effect. In contrast, for the aromatics, the products often have better ordering, but the pressures required are much higher. To synthesize ordered polymeric materials via topochemical PIP at relatively low pressure, we investigated the PIP of 1,4-diphenylbutadiyne (DPB), aiming to take the advantage of both aryl and ethynyl [65]. We obtained crystalline armchair graphitic nanoribbons, and, importantly, revealed a new PIP route, dehydro-Diels−Alder (DDA) reaction, which conforms exactly to the topochemical strategy. Figure 7a displays the in situ XRD patterns under HP. Before chemical reactions, DPB maintained the low-pressure structure (monoclinic phase). The crystal structure at the reaction threshold pressure of 10 GPa showed that the intermolecular distance 3.22 Å (between the alkynyl and phenyl group) and 3.24 Å (between phenyl groups) were both much smaller than those between diynes, where the shortest distance is 4.29 Å, and is still greater than the commonly required distance (4 Å) for 1,4-addition polymerization of diynes at ambient conditions (Figure 7b). It is the aryl groups that provide a large steric hindrance to prevent the direct addition reaction of triple bonds from approaching and bonding, and hence avoid the possible 1,4-addition product. In such a local structure, the new reaction path, [4+2] DDA reaction between phenyl and alkyne groups, is activated (Figure 7c), and results in the formation of a graphitic ribbon structure.

**Figure 8:**
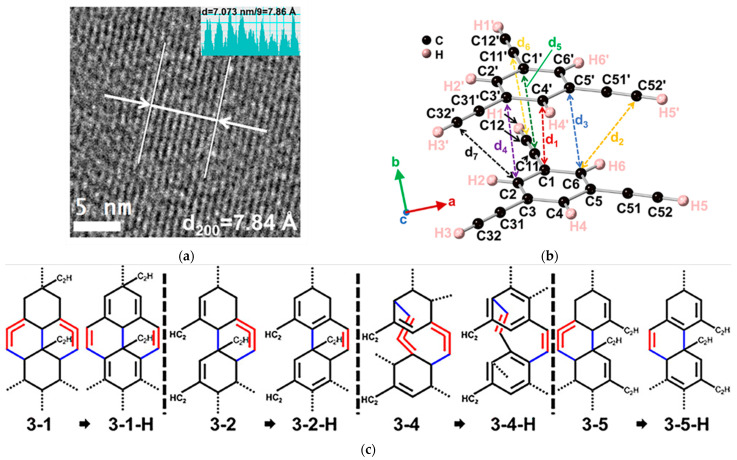
(**a**) High-resolution transmission electron microscope (TEM) of the recovered sample PE5, which were synthesized at 5 GPa using PE Press; (**b**) Crystal structure of TEB optimized at 3.6 GPa. The close intermolecular C⋯C distances are d_1_ (3.39 Å), d_2_ (3.89 Å), d_3,4,5_ (3.30 Å), d_6_ (3.33 Å) and d_7_ (4.10 Å); (**c**) Models of ribbons (3-1, 3-1-H, 3-2, 3-2-H, 3-4, 3-4-H, 3-5 and 3-5-H). New bonds are in blue, and double bonds are in red. Reprinted with permission from ref. [66]. Copyright 2021 American Chemical Society.

Subsequently, a similar DDA reaction was also observed for 1,3,5-triethynylbenzene (TEB), where three ethynyl groups were located in the meta-position of a phenyl ring [66]. Under compression, a one-dimensional nanoribbon structure containing sp, sp^2^, and sp^3^ carbons was obtained (Figure 8a). Through in situ spectroscopic analysis, we found that the phenyl moiety and the ethynyl groups reacted synergistically at a low pressure of 4 GPa. As displayed in Figure 8b, the crystal structure at 3.6 GPa determined by in situ XRD revealed that the D_C_ for PIP was around 3.3 Å, longer than that of aromatics (2.8 Å) and even alkynes (3.1 Å), but close to the distance of the forementioned DPB case (3.24 Å). Accompanied by the structure by Rietveld refinement and NMR experimental data, the theoretical simulation of PIP process proposed reasonable product models, most of which were formed via the intermolecular [4+2] DDA reaction route between the ethynylphenyl groups and the phenyl ring (Figure 8c). Although the original location of the involved ethynyl groups is relatively far from the benzene ring (d_2_ = 3.89 Å and d_7_ = 4.10 Å), the tendency of forming six-member rings is still favored, which is supposedly due to the flexibility of the terminal ethynyl, in contrast to the restrained alkynyl moieties in DPB. Moreover, it also could reasonably explain the remarkable decrease in the reaction pressure.

In summary of the addition polymerization via PIP, we can conclude the critical reaction distance rule for the PIP of specific unsaturated functional groups. When approaching a certain distance (~3.0 Å), unsaturated atoms generally tend to bond. The critical distance rule greatly helps us to design suitable precursors for synthesizing novel carbon materials via PIP. As presented Table 1, there are still several factors influencing the critical distance, including the functional groups, reaction element types, and electron charge numbers. The D_C_ of pure aromatics (2.8 Å) is relatively shorter than that of alkynes (3.1 Å), which means that more compression is required to initiate the reaction. Aromatics always have extra stability but worse reactivity. When the ethynyl groups are introduced into a benzene ring, the delocalized conjugation is expanded spatially, and the benzene ring is “activated” by the ethynyl. When the local structure allows, a synergistical reaction [4+2] is preferred with longer D_C_. It seems that the synergistical bonding naturally has longer D_C_, since the two bonding can promote each other. In addition, comparing DPB with TEB, the latter molecule has a terminal ethynyl group, and the D_C_ is accordingly increased, probably since the terminal ethynyl has more dynamic freedom, while for the metal carbide, as the charge of C_2_^2−^ caused strong electrostatic repulsion, it is harder to be activated, and a smaller D_C_ is required for triggering reactions.

### 2.2. PIP: Condensation Reaction with Hydrogen Transfer

Condensation means the production of polymers together with small molecules. Roughly speaking, this is not preferred, similar to the addition reactions under HP, since the volume shrinkage is not significant. However, some pressure-induced condensation polymerizations are often reported. For example, the hydrocarbons are often reported to transform to graphite or diamond, with a loss of hydrogen [67,68,69,70]. This is a decomposition process in the view of composition, but also a condensation polymerization process in the view of structure. At times, hydrogen may leak from the HP sample environment to the gasket material or ambient environment, which promotes the condensation process in a thermodynamical view, and was discussed in our recent work [4]. Owing to the excellent dynamics of hydrogen under HP, the transfer and elimination of hydrogen-bearing molecules is likely the most important step in the condensation polymerization under HP.

#### 2.2.1. Alkane

Alkane is highly inert and difficult to employ for polymerization since all the atoms are saturated by the C-H sigma bond. However, under natural conditions, the synthesis of heavy hydrocarbons or hydrogen-carbon polymers could occur under HPHT conditions without a catalyst on a planet or on Earth. Experimentally, by using the laser-heating diamond anvil cells (DAC) up to 2 GPa and 1000–1500 K, methane (CH_4_) was demonstrated to produce advanced alkanes (C_2_~C_4_), molecular hydrogen, unsaturated hydrocarbon, and graphite [67]. Moreover, methane decomposes to form diamonds at 10–50 GPa and 2000–3000 K [68]. At 48 GPa, methane dissociates at 1200–1500 K with the formation of hydrogen, and it decomposes further to form large amounts of solid carbon, hydrogen, and higher hydrocarbons when heating above 1500 K [69]. From the view of the reaction mechanism, the removal of hydrogen atom from alkane was the initial step under HPHT, and the remaining carbon-rich part tended to link together and then form larger molecules with thermodynamical stability. This process could be regarded as the polymerization of alkane, and the fundamental principle is the condensation reaction with the elimination of molecular hydrogen (H_2_).

**Figure 9:**
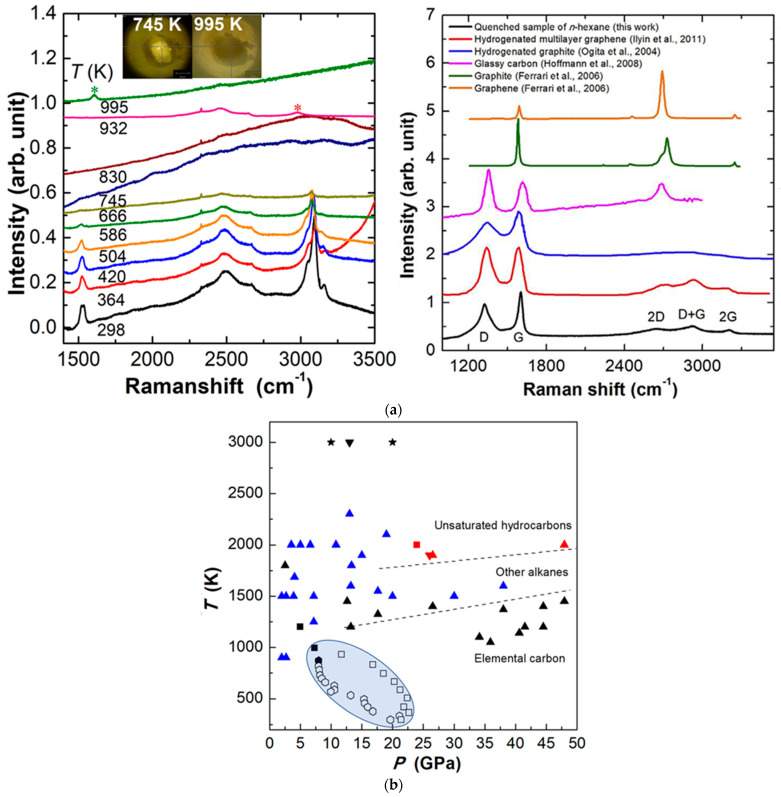
(**a**) Selected Raman spectra of n-hexane with increasing temperature at 21.4 GPa. The new peaks are marked by the asterisks (**left**); Raman spectra of quenched sample outside DAC (**right**); (**b**) C-H system experiment results under HPHT with different staring materials. Reprinted with permission from ref. [70]. Copyright 2021 Elsevier.

Recently, we explored n-hexane and cyclohexane under extreme conditions. Under high pressure and room temperature, both n-hexane and cyclohexane only underwent reversible phase transitions up to approximately 42 GPa without any chemical reaction [70]. By using resistive heating combined with DAC, the dehydrogenation reactions and polymerization under HPHT proceed for both n-hexane and cyclohexane. For n-hexane under 21.4 GPa, when heated to 745 K, intense fluorescence appeared, indicating the formation of unsaturated compound with conjugated structures such as aromatics or conjugated alkenes. At 932 K, new alkanes were formed as new peaks appearing in the region of sp^3^-CH. At 995 K, an extended carbon structure was formed and determined to be a partially hydrogenated graphite (Figure 9a). The whole process was the dehydrogenation of alkanes, formation of conjugated hydrocarbons, and subsequently the production of polymeric carbon-based species. For cyclohexane, unlike n-hexane, no strong fluorescence was observed during the heating process, indicating a different reaction pathway, which still needs further investigation, whereas the final products synthesized from cyclohexane were very similar to that of n-hexane, which means the hydrogen transfer reaction and condensation process also dominated under the HPHT conditions [70]. Some reported results of alkanes under HPHT are summarized in Figure 9b.

#### 2.2.2. Nitriles

**Figure 10:**
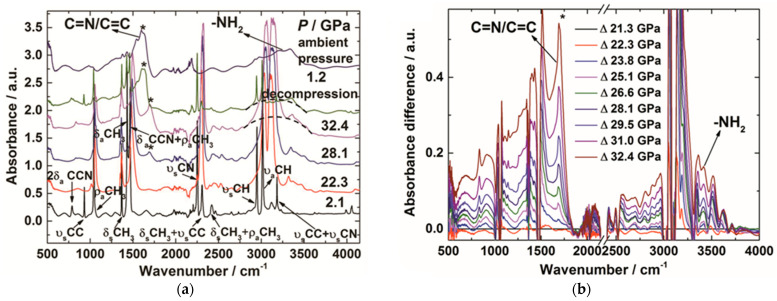

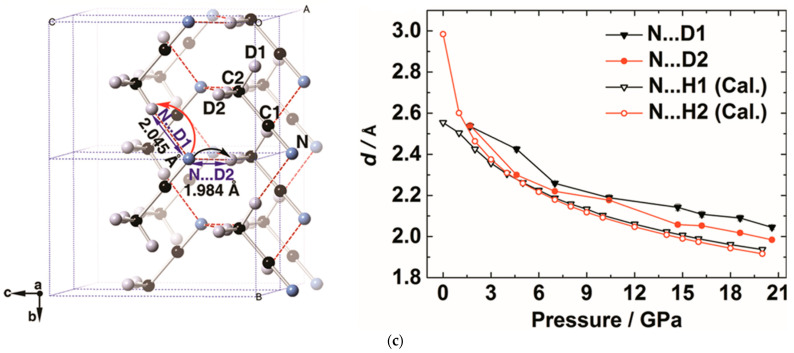
(**a**) Selected in situ IR patterns of CH_3_CN at different pressures. The asterisks display the presence of C=N/C=C group, indicating the PIP of CH_3_CN; (**b**) Selected IR spectra subtracted by the pattern of 21.3 GPa. The increasing absorbance of the C=N/C=C group is marked by the asterisks; (**c**) The crystal structure of CD_3_CN at 20.6 GPa and the evolution of selected intermolecular atomic distances as a function of pressure. N···D1 and N···D2 represent the corresponding intermolecular atomic distances obtained from the neutron diffraction data; N···H1 (Cal.) and N···H2 (Cal.) represent the distance obtained from DFT calculations. Reprinted with permission from ref. [14]. Copyright 2016 Angewandte Chemie International Edition.

The condensation polymerization via hydrogen transfer process was also observed under HPRT conditions. Acetonitrile (CH_3_CN) is a prototype of nitriles, and widely used as a reaction solvent for its stability. Its C-H bond is too stable to be activated. However, in our study, the C-H bond of acetonitrile was activated by cyano groups at high pressure and room temperature [14]. In situ mid-IR spectra of acetonitrile demonstrated that the C=N/C=C and amino groups emerged at 23 GPa and maintained when releasing to ambient pressure (Figure 10a,b). This clearly indicated that the cyano groups underwent a hydrogen transfer process, from CH_3_ to C≡N along the CH∙∙∙N hydrogen bond, and generated amino groups. Similar phenomena have also been reported in hydrogen cyanide under high pressure [71].

This reaction route was clearly uncovered by in situ neutron powder diffraction, by which we determined the crystal structures at the reaction threshold pressure of 20.6 GPa. The intermolecular distances between N∙∙∙H(D) are compressed to 2.0 Å, which was consistent with the density functional theory (DFT) calculation results (Figure 10c). This D_C_ is significantly shorter than the sum of van der Waals radii (2.75 Å) of H and N by ~27%. Since the N-H bond length was approximately 1 Å and the H atom only needs to move 1 Å to transfer, considering the thermal vibration and the tunneling effect, we concluded that under HP the hydrogen transfer from -CH_3_ to -CN along the hydrogen bond, as indicated by the arrows in Figure 10c.

In contrast to the alkanes, acetonitrile is unsaturated. During the hydrogen transfer process, active intermediates (possibly similar to CH_2_=C=NH and CH≡C-NH_2_) was produced and the polymerization reaction (C-C bonding) was triggered simultaneously. This is thermodynamically preferred, and promoted the hydrogen transfer process. By using DAC as high-pressure generation apparatus, we obtained a yellow product containing sp^2^-, sp^3^-carbon, imine (-CH-C=NH), and amino groups(-NH_2_). When using PE press which has a relatively larger pressure gradient and easily cause cracks in the gasket, an obvious releasing of ammonia gas was detected, and the rest of the black product was found to be a graphitic polymer. Therefore, the whole polymerization process leading to the graphitic polymer can be regarded as a consecutive condensation reaction, accompanied with the loss of NH_3_. Similar condensation polymerizations were also reported in other nitrogen-containing molecules, for instance octahydro-1,3,5,7-tetranitro-1,3,5,7-tetrazocine (HMX) which has nitro groups, with the high temperature condition needed for activation [72].

With respect to the reason of small molecule loss, we conclude the pressure gradient is a significant factor to accelerate hydrogen transfer dynamically, which has been observed in several systems experimentally and discussed theoretically. For example, in the forementioned acetylene and acetonitrile, when the sample was not well sealed or obvious gasket breaking appeared, a more conjugated graphene-like structure was formed in the “unsuccessful” experiments. For the HPHT experiments in alkanes, heating will also easily result in some cracks in the gaskets. In these situations, a large pressure gradient would be formed, which could promote the leak of small molecules produced from the hydrogen transfer reaction. As the reaction proceeds, the elimination of small molecules would conversely promote the hydrogen transfer reaction and lead to the formation of an extended carbon-base framework under high pressure.

## 3. Characterization Methods

For high-pressure investigation, typical characterization methods include in situ Raman and IR spectroscopy, XRD, neutron diffraction, and PDF and XAFS, as well as ex situ analysis methods such as liquid/solid NMR, mass spectrometry, SEM and TEM, etc. Herein, we hope to give an emphasis on neutron diffraction and mass spectrometry as their unique advantages on analyzing the organic systems and chemical reactions.

### 3.1. In Situ Neutron Diffraction under High Pressure

For exploring crystalline structures under high pressure, the most widely used method is single crystal/powder XRD technology. However, in most of reported high-pressure cases, hydrogen is basically invisible by X-ray due to its low coherent scattering cross section. For neutron diffraction, since neutrons interact directly with nuclei, there is a high sensitivity to low Z atoms, such as C, H, N, O, etc. It has been proven to be particularly suitable for distinguishing the positions of light elements and the adjacent elements in the crystal structure, and has unique advantages in studying hydrogen transfer phenomena in organic molecules. For example, in the high-pressure experiment of acetonitrile, the position of hydrogen atoms was effectively determined by neutron diffraction [14]. Until recently, neutron diffraction at high pressure has realized the measurement up to above 25 GPa using opposed toroidal anvils for routine measurement [73,74] and above 90 GPa using opposed diamond anvil devices [75,76], both of which could basically satisfy the demand of organic compounds and implement the crystal structure determination before reactions.

Unlike XRD, only very few in situ HP neutron diffractometers are available in the world, including constant wave diffractometer [77] and time-of-flight diffractometer [78,79,80,81,82,83]. The former can cover large d-spacing when using opposed anvil devices (such as PE press) or piston-cylinder press, both of which have enough 2θ openings. The latter mainly use the data at around 2θ = 90^o^, hence do not require large opening on the HP devices. The instruments also have different resolution and intensity distributions, etc. It is necessary to consider what kind of data is needed and the feature of the instrument before the experiment.

Even if the instrument is suitable and in good condition, due to the small volume of the sample and the stress under HP, the neutron diffraction data often have limited quality and only at very few pressure points due to the beamtime. X-ray diffraction and theoretical optimization are often needed to evident each other, and restraining bond length/bond angle inside molecules are popular procedures. There are also some experiential tricks for data analysis. For example, sometimes the diffraction patterns look the same with only slight shift in d-spacing. Then, the refinement most likely results in the same Bragg index for a special peak in different patterns. Failure in fitting the peaks using the same set of Bragg indexes (lattice parameters) across the pressure range may suggest a phase transition, or the lattice parameters are wrong. When refining diffraction data with good quality, we often perform Le Bail fitting (or Pawley fitting) to obtain lattice parameters first, and then Rietveld refinement with the atomic positions. Due to the significant peak overlap and the background/noise, sometimes we must refine lattice parameters with fixed atomic positions, since the latter would provide intensities to the peaks, which would facilitate the refinement of lattice parameters.

### 3.2. High Resolution Gas-Chromatography-Mass-Spectrometry

In complement to diffraction, the techniques that can probe the local structures of the molecules/materials are of great importance to understand the chemical reactions under HP. The gas-chromatography-mass-spectrometry (GC-MS) is a fundamental analytical tool for composition separation and chemical identification. Compared to other technologies such as normal spectroscopy and total-scattering (pair distribution function) [84], the featured advantage of GC-MS is the separation of components to then analyze them one-by-one. The MS can give the exact molecular formula of the recovered components and their fragments. In the research of metal carbide, the GC-MS experiments show solid evidence of PIP of C_2_^2−^ moiety, though the polymeric structure without long range order could not be analyzed by diffraction methods [31,32].

Besides, it is found that several different reaction pathways often coexist in high-pressure reactions, which results in complicated products with huge content differences. Utilizing the chromatography method, especially the capillary columns, we could efficiently separate the great number of components according to their distinguishing characters, such as molecular weight, boiling points, polarity, molecule size, and so on. In the PIP work of C_6_H_6_-C_6_F_6_ co-crystal, over one hundred of molecules were detected using gas chromatography-mass spectroscopy (GC-MS) [58]. By comparing the relative percentage of every component in the PIP products recovered from different pressures, we could figure out the key component in PIP process of aromatics.

Furthermore, in conjunction with high resolution MS, we could obtain accurate mass, certain formula, and the fragment patterns [85]. Based on these, it is possible to deduce the molecular structure, to intuitively understand the bonding information from the molecular monomer to the polymer under high pressure. In addition, MS can have a sufficiently high sensitivity, which enables to study the sample in submicrogram quantity obtained from DAC, which greatly expands the high-pressure research scope. In order to better collect sample and minimize loss as well as prevent external pollution, our group has made a great attempt to develop sampling methods and apparatuses to match and cooperate high pressure generation devices, which has been successfully conducted in several high-pressure research [31,32,58].

## 4. Conclusions

In this paper, we have reviewed the pressure-induced polymerization (PIP) of organic compounds and summarized the classification of polymerization, which is mainly divided into addition polymerization and condensation polymerization. The addition polymerization occurs in unsaturated monomers, such as alkynes and aromatics. There is a certain critical reaction distance in PIP. For example, when the closest intermolecular carbon…carbon distance between alkynes approach ~3.0 Å, the addition reaction generally starts. Further investigation illustrated that there are several factors that influences the value of D_C_, including the type of unsaturated groups, the quantity of electric charge and reaction route. Compared to aromatics with a more confined lattice motion, alkynes are more easily activated with a longer D_C_. Synergistic reactions such as [4+2] addition also has longer D_C_. Furthermore, in comparison to the substituted phenyl groups of DPB, TEB has a terminal ethynyl pendant, which is more flexible in approaching the neighbored molecules and efficiently initiating chemical reactions, and hence has a longer D_C_, while for the metal carbide, the strong electrostatic repulsion of C_2_^2−^ requires a shorter D_C_ for reaction. For condensation polymerization under HP, it is interesting to note that the involved types of reaction monomers are diverse, including saturated and unsaturated molecules. The PIP is accompanied with elimination of the small molecules, which implies the occurrence of hydrogen transfer reaction in the PIP. Hydrogen bond acts an important role for initiating the hydrogen transfer reaction and activating the PIP. Moreover, pressure gradient is also inferred to be a driving force to promote the condensation reactions, by the means of continuously releasing the small molecules and pushing on the polymerization of unsaturated carbons under high pressure.

By comparing with the solution reaction activated by heating, the pressure-induced reaction seems highly “controllable”, the molecules can be manually pushed to some certain threshold to bond/debond step-by-step. This provides a very valuable opportunity to follow and probe the reaction process in a “static mode”, not only meaningful for HP reaction, but also for understanding other reactions. For example, the measurement and understanding of D_C_ is still at the very beginning stage, but it may provide important insight to the research of reaction under ambient conditions. In the future, more reactions and rules under HP can be expected. This would contribute to another important topic, the selectivity of the reactions. We did not focus on this in the review. Unlike the molecular compounds, for the synthesis of materials with extended covalent bonded structures, it is difficult to remove the unwanted “impurity” once it is produced, and it is also not always possible to extend the reaction time or heat up, since the target material is often metastable. Therefore, we need to improve the reaction selectivity to satisfy the requirement of the purity. This again requires us to understand more chemical reactions, including the process, mechanism, and conditions, etc. When we accumulate a large toolbox and have better control of the reaction condition, more complex covalent-bonded structures can be designed and synthesized. Following a retro-synthetic rule and under the help of theoretical calculation, we believe most of the dynamic stable structures can be synthesized sooner or later, similar to what is currently happening in organic chemistry.

## Figures and Tables

**Table 1:** Intermolecular distance threshold for PIP of unsaturated compounds.

Compounds	D_C_ (Å)	Reaction at Initiation	Pressure (GPa)
Acetylene (C_2_H_2_)	3.1 [23]	free radical reaction	5.7
Sodium Monoacetylene (NaHC_2_)	2.9 [34]		11
Calcium Acetylene (CaC_2_)	2.9 [31]		20
Lithium Acetylene (Li_2_C_2_)	-		33
Benzene (C_6_H_6_)	2.8 [59]	Diels-Alder/1-1′ coupling, etc. reactions	18
Benzene-hexafluorobenzene (C_6_H_6_-C_6_F_6_)	2.8 [58]		20
1,4-diphenylbutadiyne (DPB)	3.2 [65]	Diels-Alder reaction	10
1,3,5-Triethynylbenzene (TEB)	3.3 [66]		4
